# Supplementary material for: Effects of government policies and the Nowruz holidays on confirmed COVID-19 cases in Iran: An intervention time series analysis
Source: PLoS One. 2021 Aug 19;16(8):e0256516. doi: 10.1371/journal.pone.0256516 (PMC8376046; doi:10.1371/journal.pone.0256516)
Supplement: Attachment — Submitted filename: Response to Reviewers..docx [file pone.0256516.s003.docx]

Professor. Siew Ann Cheong

Academic Editor

PLOS ONE

SUBJECT: Resubmission of manuscript PONE-D-20-30409R1

Re: Effects of government policies and the Nowruz holiday on confirmed COVID-19 cases in Iran: An intervention time series analysis

Dear Editor,

We would like to thank you for the attention brought to our manuscript for this second review. Also, we would like to thank the academic editor and the reviewers for their careful reading of our manuscript and their insightful comments. We have reviewed the comments and, then, reviewed the manuscript accordingly. The comments have been noted and we have done our best to revise the paper. Below is a point-by-point response to each comment raised by the academic editor and the reviewers, especially the third reviewer (original comments from reviewers appear in red, responses in black). It should be noted that the modifications in the manuscript are highlighted using track changes in Word.

We hope that the revised version of our manuscript will now meet the PLOS ONE’s publication criteria.

Yours sincerely,

Vahid Fakoor

Associate Professor, Department of Statistics, Faculty of Mathematical Sciences, Ferdowsi University of Mashhad, Mashhad-Iran,

P.O.Box, 9177943369

Mobile: 0989151038414

E-mail: fakoor@um.ac.ir

Journal Requirements:

Please review your reference list to ensure that it is complete and correct. If you have cited papers that have been retracted, please include the rationale for doing so in the manuscript text, or remove these references and replace them with relevant current references. Any changes to the reference list should be mentioned in the rebuttal letter that accompanies your revised manuscript. If you need to cite a retracted article, indicate the article’s retracted status in the References list and also include a citation and full reference for the retraction notice.

ANSWER: Thanks for your comment. We have checked and modified some references (for example 2, 3, 4, 5, 6, 11, 15, 30). Also as you mentioned a paper, that is retracted, is replace with another relevant reference as bellow (in lines 60-63):

11.	Zareie B, Roshani A, Mansournia MA, Rasouli MA, Moradi G. A Model for COVID-19 Prediction in Iran Based on China Parameters. Arch Iran Med. 2020 Apr 1;23(4):244-248. doi: 10.34172/aim.2020.05.

1) Reviewer #3: 1. Before proceeding to the rigorous analyses of time series data, the authors need to conduct the basic statistical analysis of the data mentioning the minimum and maximum values, mean, skewness, kurtosis, etc. A diagrammatic representation of the features of the data be made (If possible).

ANSWER: We appreciate the reviewer's comments. We amended them in table 1 in the section method in page 5.

2) The authors should provide autocorrelation function (ACF) and partial autocorrelation function (PACF) plots for the time series under study.

ANSWER: Thank you for your comment. We amended autocorrelation function (ACF) and partial autocorrelation function (PACF) for the residuals of model in page 13, as required.

3) ARIMA (Or SARIMA) modeling methodology be briefly explained in the methods.

ANSWER: As you suggest, a paragraph explaining these method have been added on page 6 for more clarification on SARIMA modeling as bellow:

1. SARIMA, firstly, proposed by Box and Jenkins in the 1970s. It is presented as SARIMA (p, d, q)(P, D, Q)S, where p is the order of auto-regressive (AR), q is the order of moving average (MA), d is the order of the differences. The ACF and PACF are used for knowing the order of AR and MA. Also, based on the trend and season of time series, the order of differences will be recognized. Moreover, P, D, and Q are the corresponding seasonal orders, with S as the steps of the seasonal differences (E.P. George, G.M. Jenkins Time series analysis forecasting and control Holden-Day (1976)).

4) In the Table1, the test-statistic (e.g. t-test or Chi-square test) used need to clearly stated to find the 95% confidence intervals.

ANSWER: It should be mentioned that the statistic for obtaining 95% confidence intervals is based on Z test on page 10 and a sentence was added in this regard. A note as also been added under Table 2 (the table number is now 2 with the addition of a new table in the manuscript).

5) Could there be any future predictions using the proposed model? It would be best if the proposed model had been capable of drawing future predictions.

ANSWER: The capability of SARIMA modeling is in both forecasting and interventional analysis. So, the bellow sentences and table 3 are put in page 13.

“Table 3 shows the prediction of Covid-19 new confirmed cases, with 95 % confidence interval, in 7 days. It should be noted that the prediction can be utilized if the observed spreading pattern, and the number and type of test for detection the COVID-19 cases continues as before and if policies and restrictions are not removed. Therefore, the number of daily new cases would be 813 and 814 for 3 May 2020 and 4 May 2020, respectively.”
